# Impact of mutations in epigenetic modifiers in acute myeloid leukemia: A systematic review and meta-analysis

**DOI:** 10.3389/fonc.2022.967657

**Published:** 2022-11-28

**Authors:** Fatma Al-Bulushi, Rahma Al-Riyami, Zainab Al-Housni, Bushra Al-Abri, Murtadha Al-Khabori

**Affiliations:** ^1^ Hematopathology, Oman Medical Specialty Board, Muscat, Oman; ^2^ Hematology Department, Sultan Qaboos University Hospital, Muscat, Oman; ^3^ Internal Medicine, Oman Medical Specialty Board, Muscat, Oman; ^4^ College of Medicine and Health Sciences, Sultan Qaboos University, Muscat, Oman

**Keywords:** AML, epigenetics, mutation, ASXL1, Dnmt3a, IDH, TET2, review – systematic

## Abstract

This is a systematic review and meta-analysis evaluating the prognostic significance of epigenetic mutations on the overall survival (OS) in Acute Myeloid Leukemia (AML). We searched for studies evaluating epigenetic mutations in AML (up to November 2018) in PubMed, Trip database and Cochrane library. Hazard ratio (HR) of outcomes were extracted, and random-effects model was used to pool the results. A total of 10,002 citations were retrieved from the search strategy; 42 articles were identified for the meta-analysis (ASXL1 = 7, TET2 = 8, DNMT3A = 12, IDH =15), with fair to good-quality studies. The pooled HR was 1.88 (95% CI: 1.49−2.36) for ASXL1 mutation, 1.39 (95% CI: 1.18−1.63) for TET2 mutation, 1.35 (95% CI 1.16-1.56) for DNMT3a and 1.54 (95% CI: 1.15-2.06) for IDH mutation. However, there was a substantial heterogeneity in the DNMT3a and IDH studies. In conclusion epigenetic mutations in ASXL1, TET2, DNMT3a and IDH adversely impact OS in patients with AML albeit with considerable heterogeneity and possibly publication bias. Further studies are required to address these limitations.

## Introduction

Acute myeloid leukemia (AML) is characterized by the uncontrolled proliferation of myeloid blast cells in the bone marrow and peripheral blood (1). Breakthroughs in the past have contributed to our understanding of the genetic failures and the changing biology in the myeloid cells that underlie the initiation and progression of the disease (2). With the application of global DNA sequencing, several recurrent gene mutations have been identified, which has led to improvements in prognostication and molecular characterization within these subsets (3).

It is now recognized that genetic and epigenetic modifications are similarly important in the pathogenesis of AML (2). Epigenetic modification refers to variability in gene expression without underlying genetic changes (4, 5). DNA methylation and histone modifications are the well-known molecular epigenetic mechanisms studied in cancer biology (4). These epigenetics affect gene expression leading to leukemogenesis through silencing tumor suppressors and activation of oncogenes (3). Multiple studies integrating epigenetic modifiers like DNMT3a, IDH1, IDH2, TET2, ASXL1 and EZH2 and clinical outcomes in AML patients identified mutations as markers prognostic stratification (1). In addition to prognostic significance, since these alterations do not change the DNA sequences and are pharmacologically reversible, they have been regarded as optimal targets for what is now known as epigenetic therapy (2).

Several studies and reviews assessed the prognostic significance of these mutations in AML patients; however, the results are widely variable. In a study performed by Ravandi et al. (6) and colleagues, IDH mutation showed no impact on response to therapy nor Overall Survival (OS). However, Shunichiro Yamaguchi and colleagues (7) found IDH mutations are associated with poor prognosis. A meta-analysis performed by Qingyu Xu and colleagues (8) included thirty-three studies and concluded that IDH1 is associated with poor prognosis and IDH2 is associated with good prognosis. Similarly, studies assessing the impact of DNMT3A, TET2, ASXL1 and EZH2 showed conflicting results. Thus, it’s necessary to perform a systematic review and meta-analysis to clarify the prognostic significance of these mutations in AML patients. The rationale of our study is to evaluate the impact of epigenetic mutations in AML patients with the inclusion of recent publications and larger sample size.

## Methods

### Search strategy

We performed a literature search on several electronic databases, including PubMed (https://pubmed.ncbi.nlm.nih.gov), Trip database (https://www.tripdatabase.com) and Cochrane library (https://www.cochranelibrary.com) up to November 2018. We used various medical subject headings (MeSH terms) and free text search like mutations, epigenetic, acute myeloid leukemia, acute myeloblastic leukemia, acute myelocytic leukemia and AML.

### Study selection

We included adult and pediatric AML studies that received any kind of therapy and were tested for epigenetic mutations (DNMT3a, IDH1, IDH2, TET2, ASXL1 and EZH2). In addition, observational and experimental studies were included. We excluded studies not published in the English language or those not reporting outcomes of interest. Two reviewers (RR and FB) independently screened all citations. Unrelated articles and duplicate publications were excluded after the title and abstract screening. Full-text articles were obtained from the remaining articles and were reviewed carefully for eligibility. Any disagreement between the two reviewers was resolved by discussion or involving a third reviewer (MK).

### Data abstraction

Data were extracted using a common data collection form which was designed specifically for this study. Two reviewers abstracted the data independently and subsequently compared the results. Any disagreement was resolved by discussion and consensuses. The main variables extracted were patient characteristics, type of therapy, cytogenetics and molecular features, Hazards ratio (HR) and 95% Confidence Interval (CI) for the OS. In all studies that did not report HR or CI, we used Parmar’s methods to estimate the HR from the available data (9). Furthermore, corresponding authors were contacted for missing data.

### Risk of bias assessment

The two reviewers assessed the quality of included studies independently using Newcastle-Ottawa-Scale (NOS) for observational studies. The NOS assessment tool included three main elements related to selection, comparability and outcome. A study can be awarded as good, fair or poor quality based on the maximum score. Any discrepancy between the reviewers was resolved by discussion. Cohen kappa coefficient (k) was calculated to assess the agreement.

### Analysis and data synthesis

All statistical analyses were performed using R Program version 3.1.2 (R Core Team (2020); R: A language and environment for statistical computing. R Foundation for Statistical Computing, Vienna, Austria. URL https://www.R-project.org/). Hazards ratio was used to assess the prognostic impact of epigenetic mutations compared to wild type. The random-effects model was used to pool the results for the HR of OS. Pooled HR less than 1 indicated better outcome among mutated patients. *A p-value* of 0.05 or less is considered statistical significance. The heterogeneity among studies was assessed by Cochran I^2^ and Chi-squared test. I-square (I^2^) < 30%, 30%–50%, 50%–75%, and >75% were defined as low, moderate, substantial, and considerable heterogeneity, respectively. No ethical approval was required for this study as all data were abstracted from published papers.

## Results

### Study selection

**Figure 1:**
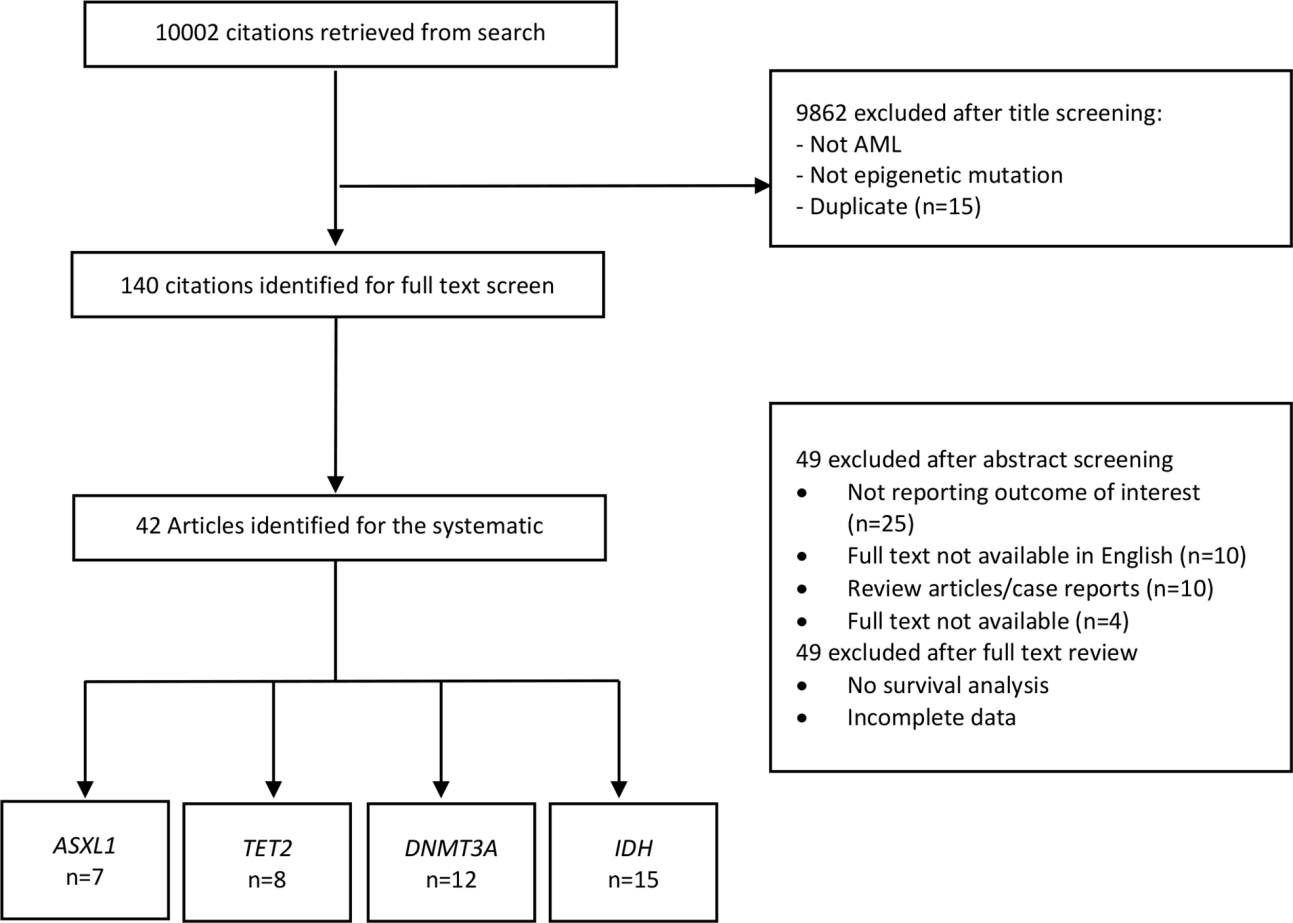
Study selection for the systematic review and meta-analysis. AML, Acute myeloid leukemia; ASXL1, Additional Sex Combs-Like 1; TET2, Ten-Eleven Translocation 2; DNMT3A, DNA Methyltransferase 3 Alpha; IDH, Isocitrate dehydrogenase.

The procedure of study selection is presented in **Figure 1**. Initially, 10,002 citations were retrieved from the database search. After title screening, 9862 articles were excluded either because not assessing epigenetic mutations, not AML patients or duplicates. We screened the remaining 140 articles. Of these, 49 were excluded after abstract screening for the following reasons: 14 full text not available, ten review articles/case reports and 25 not reporting the outcome of interest. Full-text screening further excluded 49 articles due to the absence of survival analysis or incomplete data. Finally, 42 articles were identified for the systematic review which met the full inclusion criteria. Of these, seven articles were for ASXL1 mutation (10–16), eight for TET2 (17–24), twelve for DNMT3A (25–36) and fifteen for IDH mutation (6, 7, 37–48).

### Study characteristics and risk of bias

**Table 1** presents the characteristics of the 42 included studies. For ASXL1 mutation articles, five were from Europe and two from Asia. The total number of patients included was 4115 patients; among these, 276 harbored the mutation. One article was for pediatric patients, and the remaining six articles were for adult patients. Eight articles were for TET2 mutation, with a total number of patients 3286 among these 364 had TET2 mutation. Three studies were from Asia, four from Europe and one from Europe and the USA. Finally, for DNMT3A mutation papers total of 12 articles were included, seven from Europe, four from the USA and one from Asia, with a total number of patients 5555 and 1324 with the mutation.

**Table 1:** Description of the studies included in the systematic review.

First Author	Year	Region	Mutation	Description	NOS	Total	Mutated	Age (range)	WBC (Range)	Cytogenetic abnormalitis	HR estimation
Chou WC	2010	Taiwan	ASXL1	AML	6	360	26	66	_	Favourable 8, Intermediate 17, Adverse 1, Unknown 0	HR
Pratcorona M	2012	Netherlands	ASXL1	AML	6	807	41	54 (15-74)	13 (1.1-220)	_	HR
Schnittger S	2013	Germany	ASXL1	CN-AML	5	481	51	71.8	34.2	Favourable 0, Intermediate 51, Adverse 0, Unknown 0	Survival curve
El-Sharkawi D	2014	UK	ASXL1	AML	7	367	32	61.5 (19-74)	42.75 (3-528)	Favourable 1, Intermediate 20, Adverse 2, Unknown 9	HR
Devillier R	2015	France	ASXL1	AML	5	35	14	_	_	Favourable 0, Intermediate 14, Adverse 0, Unknown 0	HR
Paschka P	2015	Germany	ASXL1	AML	6	1696	103	53 (36-61)	6.5 (0.7-126.5)	Favourable 10, Intermediate 65, Adverse 24, Unknown 4	Survival curve
Yamato G	2017	Japan	ASXL1	AML	8	369	9	9 (2.2-17.9)	6.9 (3.8-218.2)	Favourable 7, Intermediate 1, Adverse 0, Unknown 1	Survival curve
Chou WC	2011	Taiwan	TET2	AML	6	486	46	68 (21-90)	43.16 (1.68-277.25)	Favourable 0, Intermediate 46, Adverse 0, Unknown 0	HR
Kosmider O	2011	France	TET2	AML	4	247	49	71	20.3 (6-98.9)	Favourable 0, Intermediate 25, Adverse 15, Unknown 9	HR
Metzeler KH	2011	USA/Germany	TET2	CN-AML	5	418	95	66 (20-80)	33.5 (1.6-450)	Favourable 0, Intermediate 95, Adverse 0, Unknown 0	Survival curve
Gaidzik VI	2012	Germany	TET2	AML	8	783	60	51 (19-60)	20.9 (0.8-192)	Favourable 11, Intermediate 39, Adverse 7, Unknown 3	Survival curve
Aslanyan MG	2014	Netherlands	TET2	AML	7	357	27	54 (24-59)	32.1 (1.1-240.8	Favourable 0, Intermediate 15, Adverse 1, Unknown 11	HR
Damm F	2014	Germany	TET2	CN-AML	7	215	13	53 (38-59)	32.1 (1.4-98.8)	Favourable 0, Intermediate 13, Adverse 0, Unknown 0	HR
Tian X	2014	China	TET2	CN-AML	6	373	60	50 (16-83)	78.6 (1.3-397)	Favourable 0, Intermediate 60, Adverse 0, Unknown 0	Survival curve
Ahn JS	2015	Korea	TET2	CN-AML	5	407	14	_	_	Favourable 0, Intermediate 14, Adverse 0, Unknown 0	HR
Ley TJ	2010	USA	DNMT3a	AML	6	281	62	53.1	46.4	Favourable 0, Intermediate 56, Adverse 4, Unknown 2	Survival curve
LaRochelle O	2011	France	DNMT3a	AML-IR	7	149	39	47 (20-63)	52 (1-250)	Favourable 0, Intermediate 39, Adverse 0, Unknown 0	HR
Thol F	2011	Germany	DNMT3a	AML	6	489	87	52 (30-60)	38 (0.5-328.2)	Favourable 1, Intermediate 82, Adverse 4, Unknown 0	HR
Ribeiro AF	2012	Netherlands	DNMT3a	AML	6	415	96	50.5 (18-60)	52.9 (1.1-278)	Favourable 0, Intermediate 85, Adverse 6, Unknown 5	HR
Marcucci G	2012	USA	DNMT3a	CN-AML	6	415	142	61 (22-82)	43.4 (0.9-434.1)	Favourable 0, Intermediate 142, Adverse 0, Unknown 0	HR
Renneville A	2012	France	DNMT3a	CN-AML	7	123	36	47 (23-58)	13 (1-152)	Favourable 0, Intermediate 36, Adverse 0, Unknown 0	Survival curve
Marková J	2012	Czechia	DNMT3a	AML-IR	6	226	67	_	_	Favourable 0, Intermediate 67, Adverse 0, Unknown 0	Survival curve
Hou HA	2012	Taiwan	DNMT3a	AML	6	500	70	61 (16-87)	32.49 (0.65-340.4)	Favourable 0, Intermediate 62, Adverse 4, Unknown 4	HR
Gaidzik VI	2013	Germany	DNMT3a	AML	7	1770	367	50.5 (18-60)	24.5 (0.2-532)	_	Survival curve
Ostronoff F	2013	USA	DNMT3a	AML	8	191	37	68 (57-81)	37	Favourable 0, Intermediate 24, Adverse 1, Unknown 12	HR
Gale RE	2015	UK	DNMT3a	AML-IR	6	914	272	48 (18-67)	37.3 (0.7-439)	Favourable 0, Intermediate 272, Adverse 0, Unknown 0	Survival curve
Sehgal AR	2015	USA	DNMT3a	AML	5	152	49	54.4 (26-78)	76.56	Favourable 0, Intermediate 40, Adverse 5, Unknown 4	Survival curve
Schnittger S	2010	Germany	IDH1	AML	7	769	52	67.2 (21.8-85.8)	5 (0.3-255)	_	Survival curve
Green CL	2010	UK	IDH1	AML	8	1333	107	_	_	_	HR
Boissel N	2010	France	IDH2	CN-AML	6	205	12	57 (41-66)	3 (0.6-11)	Favourable 0, Intermediate 12, Adverse 0, Unknown 0	Survival curve
Paschka P	2010	Germany	IDH	CN-AML	8	89	29	47 (27-60)	35 (0.2-175)	Favourable 0, Intermediate 29, Adverse 0, Unknown 0	Survival curve
Abbas S	2010	Netherlands	IDH1	AML	6	743	49	50 (20-71)	48 (1-400)	_	Survival curve
Abbas S	2010	Netherlands	IDH2	AML	6	780	86	50 (18-72)	42 (18-72)	_	Survival curve
Marcucci G	2010	USA	IDH	CN-AML	6	52	14	62 (21-82)	24.6 (0.9-152.1)	Favourable 0, Intermediate 14, Adverse 0, Unknown 0	Survival curve
Damm F	2011	Germany	IDH	AML	7	459	18	_	_	_	Survival curve
Chou WC	2011	Taiwan	IDH2	AML	8	309	36	55 (19-84)	16.5 (0.65-340)	Favourable 0, Intermediate 45, Adverse 5, Unknown 4	Survival curve
Nomdedéu J	2012	Spain	IDH	CN-AML	7	120	27	58 (17-71)	23.05 (0.8-408)	Favourable 0, Intermediate 27, Adverse 0, Unknown 0	Survival curve
Ravandi F	2012	USA	IDH	AML	8	170	52	_	_	_	Survival curve
Lin J	2012	China	IDH	AML	8	114	12	_	_	_	Survival curve
Guan L	2013	China	IDH1	AML	9	315	31	41 (14-66)	56 (1.07-456)	_	Survival curve
Yamaguchi S	2014	Japan	IDH	AML	7	226	37	60 (16-82)	16.5 (1-408)	_	Survival curve
Aref S	2015	Egypt	IDH1	CN-AML	5	110	35	_	52.1	Favourable 0, Intermediate 35, Adverse 0, Unknown 0	Survival curve

NOS, Newcastle-Ottawa-Scale; WBC, White blood cells; HR, Hazards ratio; AML, Acute myeloid leukemia; CN, cytogenetically normal; ASXL1, Additional Sex Combs-Like 1; TET2, Ten-Eleven Translocation 2; DNMT3A, DNA Methyltransferase 3 Alpha; IDH, Isocitrate dehydrogenase.

Furthermore, IDH mutation papers were 15 articles. The total number of patients was 5794, and mutated was 597. Eight papers were from Europe, four from Asia, two from the USA and one from Africa. NOS for all 42 included studies ranged from fair to good quality. Cohen kappa coefficient (k) ranged from 0 - 1, with the lowest agreement in exposure and duration of follow-up and highest agreement in confounders.

The details of chemotherapy protocol used in each publication are summarized in **Supplementary Material**. Majority received intensive cytarabine and anthracycline based chemotherapy. However a subset of frail patients received less intensive chemotherapy, palliative or best supportive care.

### Overall survival

**Figure 2:**
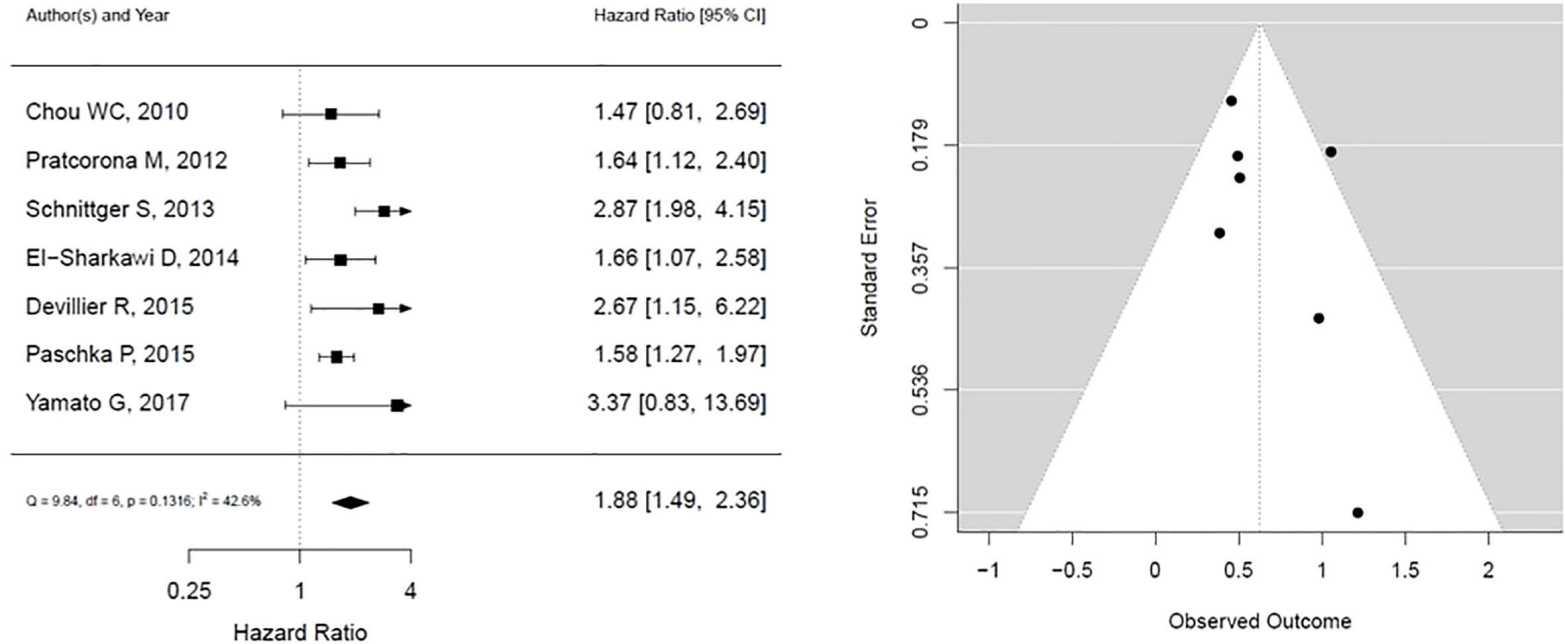
Forest plot and funnel plot of the HR for overall survival in *ASXL1* mutation. The first author and year of publication is provided for each study. The hazards ratio (boxes) with 95% confidence intervals (CI, horizontal lines) were calculated, the pooled hazards ratio (diamond) was estimated using random effect model. The P value for comparing heterogeneity between subgroups was calculated using I-squared. ASXL1, Additional Sex Combs-Like 1.

As shown in **Figure 2**, ASXL1 mutation was associated with worse overall survival (HR, 1.88; 95% CI, 1.49 – 2.36, P 0.1316; heterogeneity: I-squared 42.6%). However, this was statistically not significant and was associated with moderate heterogeneity. In addition, the funnel plot showed asymmetry suggesting publication bias or a small study effect.

**Figure 3:**
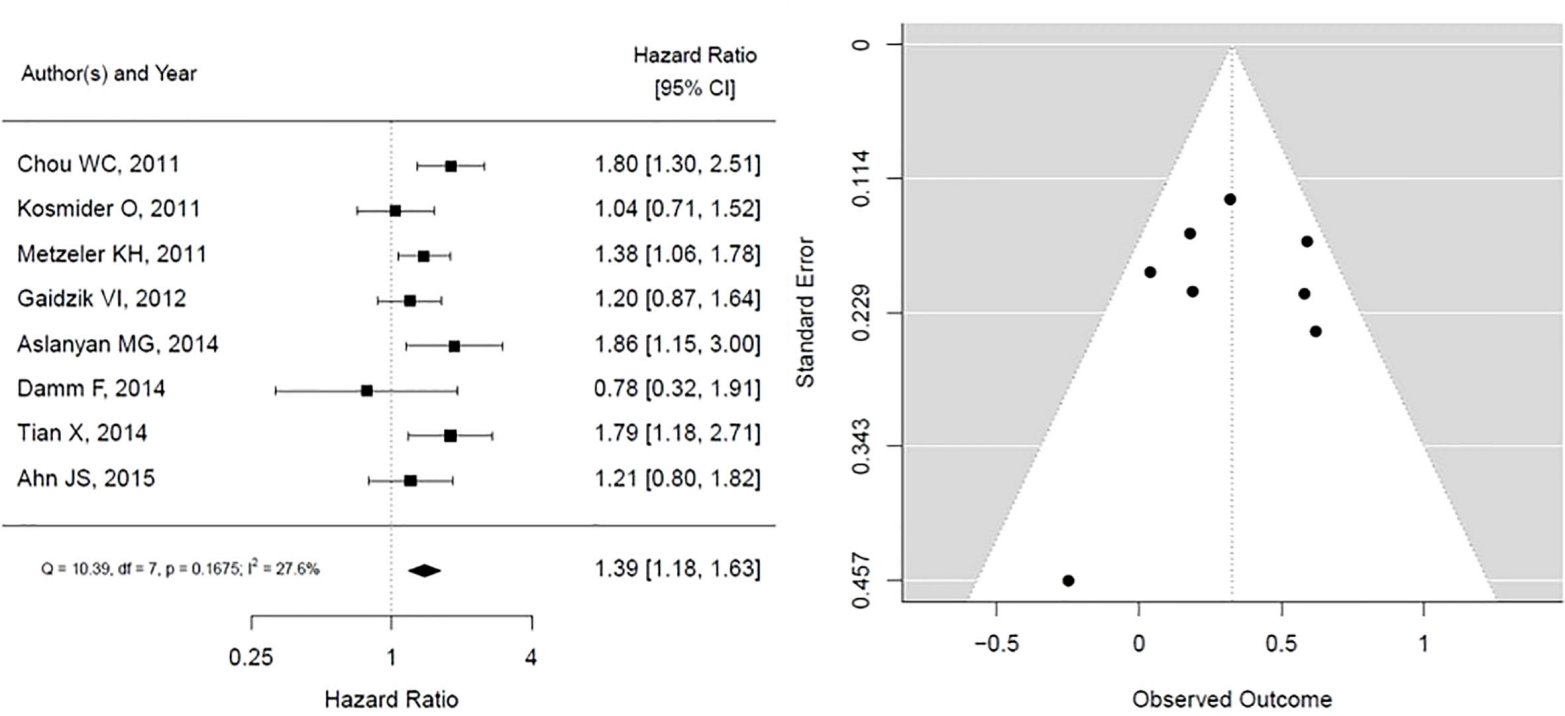
Forest plot and funnel plot of the HR for overall survival in *TET2* mutation. The first author and year of publication is provided for each study. The hazards ratio (boxes) with 95% confidence intervals (CI, horizontal lines) were calculated, the pooled hazards ratio (diamond) was estimated using random effect model. The P value for comparing heterogeneity between subgroups was calculated using I-squared. TET2, Ten-Eleven Translocation 2.

The results for TET2 mutation as presented in **Figure 3**. It shows that TET2 is associated with statistically insignificant worse overall survival with low heterogeneity (HR, 1.39; 95% CI, 1.18 – 1.63, P 0.1675; heterogeneity: I-squared 27.6%). The funnel plot is symmetrical, suggesting no publication bias.

**Figure 4:**
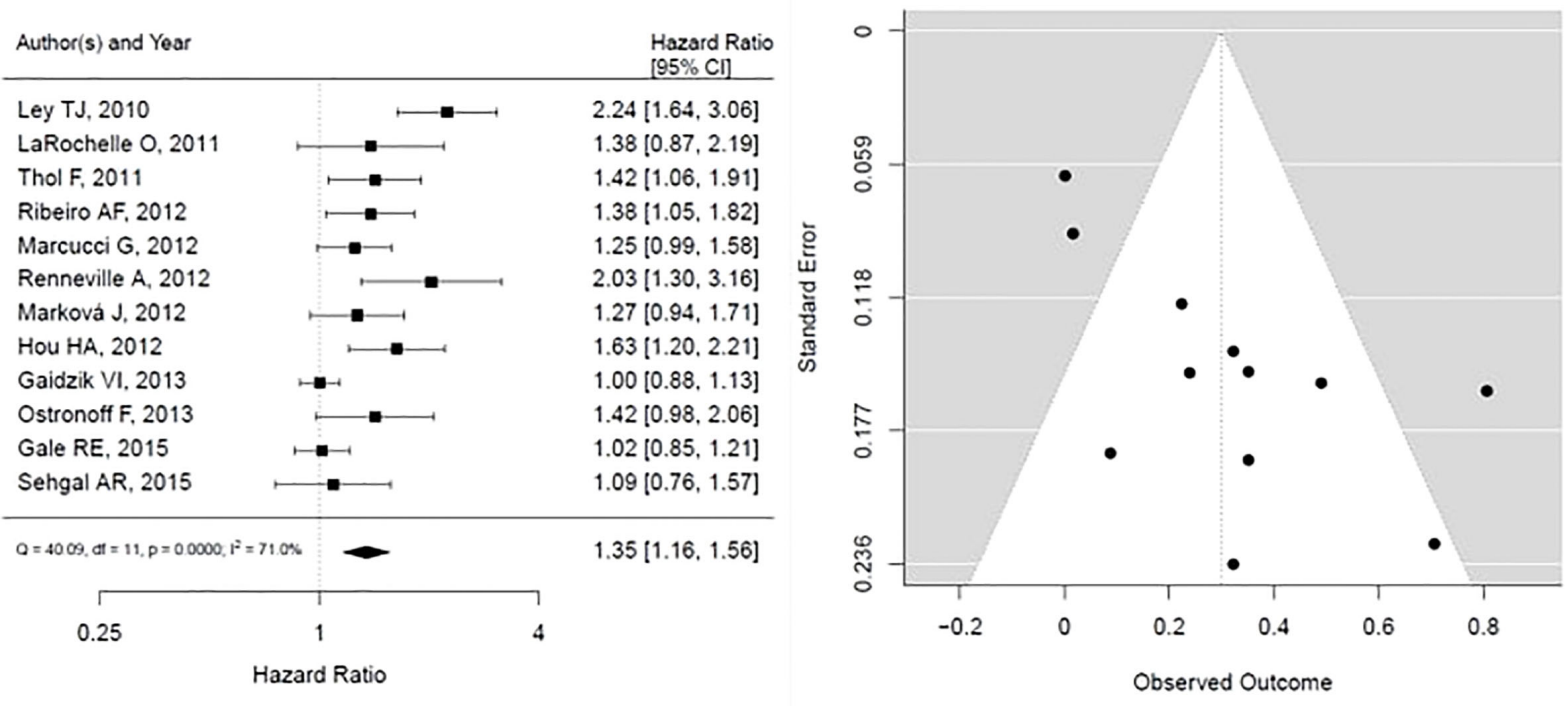
Forest plot and funnel plot of the HR for overall survival in *DNMT3a* mutation. The first author and year of publication is provided for each study. The hazards ratio (boxes) with 95% confidence intervals (CI, horizontal lines) were calculated, the pooled hazards ratio (diamond) was estimated using random effect model. The P value for comparing heterogeneity between subgroups was calculated using I-squared. DNMT3A, DNA Methyltransferase 3 Alpha.

For DNMT3A mutations, the forest plot is shown in **Figure 4**, the pooled HR is associated with worse overall survival, and the results are statistically significant, however with substantial heterogeneity (HR, 1.35; 95% CI, 1.16 – 1.56, P < 0.05; heterogeneity: I-squared 71.0%). In addition, the funnel plot is suggestive of publication bias, given the asymmetry as assessed visually.

**Figure 5:**
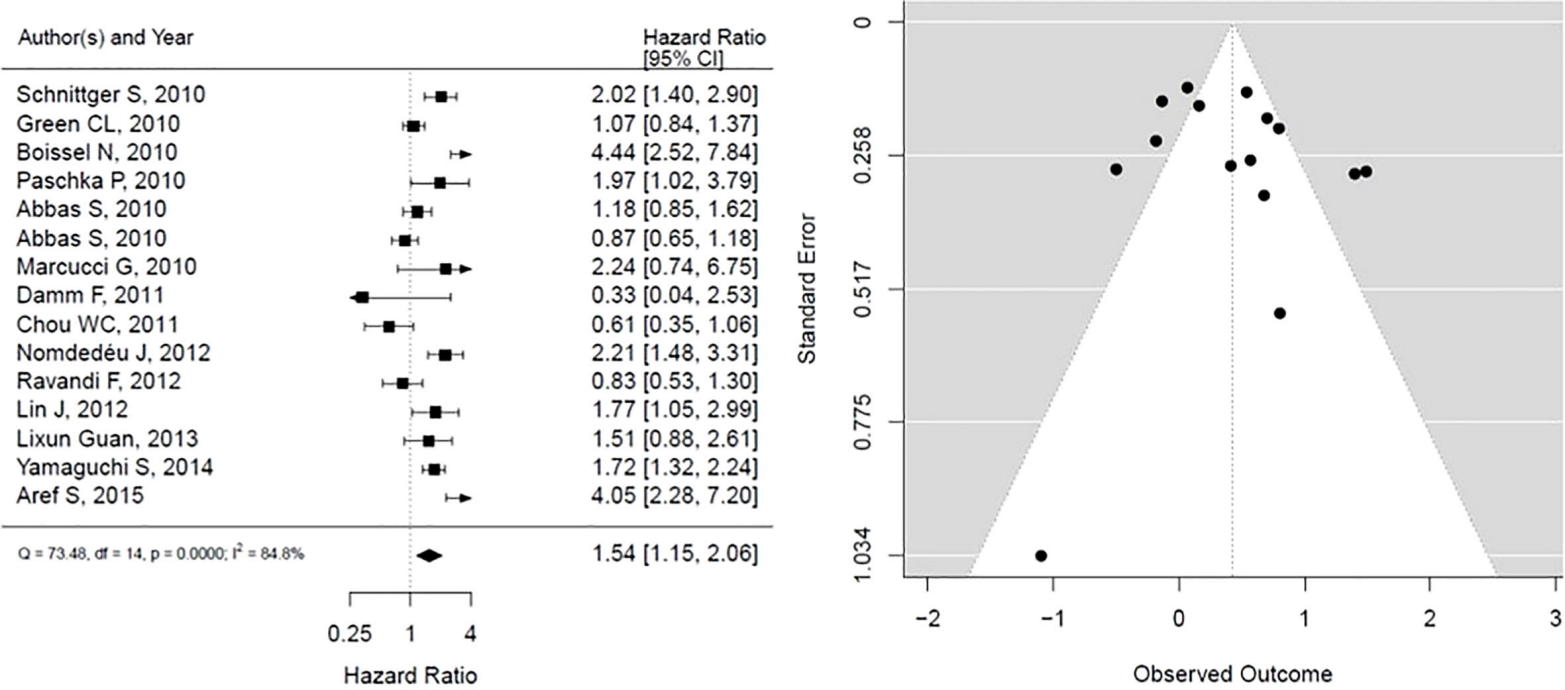
Forest plot and funnel plot of the HR for overall survival in IDH mutation. The first author and year of publication is provided for each study. The hazards ratio (boxes) with 95% confidence intervals (CI, horizontal lines) were calculated, the pooled hazards ratio (diamond) was estimated using random effect model. The P value for comparing heterogeneity between subgroups was calculated using I-squared. IDH, Isocitrate dehydrogenase.

As shown in **Figure 5**, IDH mutation is associated with statistically significant worse OS, however with considerable heterogeneity in results (HR, 1.54; 95% CI, 1.15 – 2.06, P <0.05; heterogeneity: I-squared 84.8%). The funnel plot presented is not suggestive of publication bias.

## Discussion

In this systematic review and meta-analysis, we found that all epigenetic modifiers mutations (ASXL1, TET2, DNMT3A and IDH) are associated with worse OS in patients with AML based on fair to good-quality studies. However, there was substantial heterogeneity for IDH and DNMT3A mutation studies, respectively.

For ASXL1 mutation, the results revealed a prominent worse overall survival among AML patients. This was consistent among all the included studies. Shivarov V. and his colleagues (49) reported a similar outcome based on the assessment of six large trials and a total of 3311 adult patients. Since there was a single study from the pediatric population, the results cannot be applied to this population. An adverse prognosis was also observed with TET2 mutations, consistent with what Wang R. and his colleagues (50) reported. DNMT3A mutation showed a significantly worse prognosis than the wild type, consistent with what is reported in the literature (51, 52).

Similarly, our data on IDH mutation is reported to show an adverse effect on prognosis. Moreover, we found out that these mutations were frequently found in the intermediate-risk group of the international prognostic scoring system, as shown in **Table 1**. They were also associated with older patient age and higher presenting white blood cell count.

Although there was substantial heterogeneity in DNMT3A and IDH studies, this could be explained by the year of the publication, age of the patients and cytogenetic risk profile. There has been improvement in supportive care over the last few years, and with the emergence of new targeted therapies, the OS of these patients slightly improved. Jakobsen and colleagues conducted a large population based registry study and they concluded a significant temporal overall survival improvement among patient with AML since 2000. This was particularly seen among the patients aged between 50-75 years where they got curative chemotherapy and option of allogenic stem cell transplant was offered in some cases (53). Furthermore, many other factors affect response to therapy and OS, especially the presence of FLT3 mutation. Publication bias was suggested among ASXL1 and DNMT3A mutation probably; studies with no effect were not published. This was a major limitation to our estimate of the outcome of these mutations.

Our study has several limitations. First, the lack of data from many studies and the high possibility of publication bias could impact the outcome. Second, we did not perform subgroup analysis to assess the impact of the year of the publication to explain the heterogeneity, as older publications probably had worse outcomes. Finally, we did not consider individual patient data, and our analysis was based on cumulative data from different studies.

In conclusion, this meta-analysis revealed that ASXL1, TET2, DNMT3A and IDH mutations had an adverse effect on the survival of AML patients albeit with considerable heterogeneity and possibly publication bias. Further studies are required to address these limitations.

## Data availability statement

The original contributions presented in the study are included in the article/**Supplementary Material**. Datasets are available on request from the corresponding author.

## Author contributions

MA-K suggested the idea, optimized the search strategy, performed the analysis and reviewed and edited the manuscript. FA-B performed the search, extracted the information and drafted the manuscript. RA-R extracted the data and gave final approval. ZA-H and BA-A gave the final approval of the manuscript. All authors contributed to the article and approved the submitted version.

## Acknowledgments

Would like to thank Oman Medical Specialty Board (OMSB) library for providing full text articles.

## Conflict of interest

The authors declare that the research was conducted in the absence of any commercial or financial relationships that could be construed as a potential conflict of interest.

## Publisher’s note

All claims expressed in this article are solely those of the authors and do not necessarily represent those of their affiliated organizations, or those of the publisher, the editors and the reviewers. Any product that may be evaluated in this article, or claim that may be made by its manufacturer, is not guaranteed or endorsed by the publisher.

## Abbreviations

AML, Acute myeloid leukemia; ASXL1, Additional Sex Combs-Like 1; TET2, Ten-Eleven Translocation 2; DNMT3A, DNA Methyltransferase 3 Alpha; IDH, Isocitrate dehydrogenase; FLT3, Fms Related Receptor Tyrosine Kinase 3; OS, Overall survival; HR, Hazards Ratio; WBC, White Blood Cells.
