# Supplementary material for: Impact of mutations in epigenetic modifiers in acute myeloid leukemia: A systematic review and meta-analysis
Source: Front Oncol. 2022 Nov 28;12:967657. doi: 10.3389/fonc.2022.967657 (PMC9742486; doi:10.3389/fonc.2022.967657)
Supplement: Supplementary file 1 [file DataSheet_1.pdf]

Supplementary Table 1: Description of the chemotherapy protocol used in included studies.				
First Author	Year	Region	Mutation	Chemotherapy protocol
Chou et al. (10)	2010	Taiwan	ASXL1	Induction 7+3. Cytarabine 100 mg/m^2 days 1-7 (Total dose $700mg/m^2$) and idarubicin 12 mg/m^2 for 3 days (Total dose 36 mg/m^2). Consolidation 2-4 cycles HDAC 2000 mg/m^2 every 12 hours for days 1-4 total 8 doses with or without one anthracycline.
Pratcorona et al. (11)	2012	Netherlands	ASXL1	HONOV protocols HO 04, 04A, 29, 42, 42A, 43<br>HO04 and HO04 A no data<br>HO29 cytarabine 200 mg/m^2 for 7 days + idarubicin 12 mg/m^2 for 3 days<br>HO42 cytarabine 200 mg/m^2 for 7 days + idarubicin 12 mg/m^2 for 3 days or cytarabine 1000 mg/m^2 every 12 hours for 10 doses<br>HO42A cytarabine 200 mg/m^2 for 7 days + idarubicin 12 mg/m^2 for 3 days<br>HO43 (patients 61 years and above) cytarabine 200 mg/m^2 for 7 days + daunorubicin 90 mg/m^2 for 3 days vs 45 mg/m^2 for 3 days
Schnittger et al. (12)	2013	Germany	ASXL1	Intensive therapy 7+3<br>TAD (thioguanine, cytarabine and daunorubicin)<br>HAM (High dose cytarabine and mitoxantrone)
El-Sharkawi et al. (13)	2014	UK	ASXL1	UK MRC trials AML10, AML 11, AML 12<br>AML10 daunorubicin 50 mg/m^2 for three days and twice daily cytarabine for 10 days in course 1 and 8 days in course 2 plus either thioguanine or etoposide<br>AML11 randomized to 1 of 3 induction treatments for 2 courses of DAT (daunorubicin, cytarabine, and thioguanine) 3 + 10, ADE (daunorubicin, cytarabine, and etoposide) 10 + 3 + 5, or MAC (mitoxantrone-cytarabine)<br>AML12 cytarabine, daunorubicin and etoposide (ADE) versus mitoxantrone, cytarabine and etoposide (MAE), and compared standard Ara-C dose of 100 mg/m^2 b.i.d. (S-DAT) versus 200 mg/m^2 b.i.d. (H-DAT).
Devillier et al. (14)	2015	France	ASXL1	Intensive induction chemotherapy (54% of patients) no details of chemotherapy available in publication.
Paschka et al. (15)	2015	Germany	ASXL1	Treatment protocols of AMLSG<br>AML HD98A two induction cycles of idarubicin, standard-dose cytarabine, and etoposide with or without ATRA, followed by one consolidation cycle of intermediate-dose cytarabine and mitoxantrone with or without ATRA.

				AMLSG 07-04 two induction cycles of idarubicin, standard-dose cytarabine, and etoposide with or without ATRA, followed by one consolidation cycle of intermediate-dose cytarabine with or without ATRA.
Yamato et al. (16)	2017	Japan	ASXL1	AML-05 trial treatment protocol<br>Two common courses of remission induction multi-agent combination chemotherapy for all the eligible patients.<br>For Low Risk group (LR), 3 courses of intensification multi-agent combination chemotherapy.<br>For Intermediate Risk group (IR), 3 courses of intensification multi-agent combination chemotherapy.<br>For High Risk group (HR), 1 to 3 courses of intensification multi-agent combination chemotherapy followed by allogeneic hematopoietic stem cell transplantation.
Chou et al. (17)	2011	Taiwan	TET2	343 standards induction (Induction 7+3. Cytarabine 100 mg/m^2 days 1-7 (Total dose 700mg/m^2) and idarubicin 12 mg/m^2 for 3 days (Total dose 36 mg/m^2). Consolidation 2-4 cycles HDAC 2000 mg/m^2 every 12 hours for days 1-4 total 8 doses with or without one anthracycline.) 143 palliative or low dose chemotherapy
Kosmider et al. (18)	2011	France	TET2	158 received intensive cytarabine and anthracycline (no details)
Metzeler et al. (19)	2011	USA/Germany	TET2	CALGB trial – intensive cytarabine/daunorubicin based
Gaidzik et al. (20)	2012	Germany	TET2	HD98A AML HD98A two induction cycles of idarubicin, standard-dose cytarabine, and etoposide with or without ATRA, followed by one consolidation cycle of intermediate-dose cytarabine and mitoxantrone with or without ATRA.
Aslanyan et al. (21)	2014	Netherlands	TET2	AML12 cytarabine, daunorubicin and etoposide (ADE) versus mitoxantrone, cytarabine and etoposide (MAE), and compared standard Ara-C dose of 100 mg/m^2 BID. (S-DAT) versus 200 mg/m^2 BID. (H-DAT).
Damm et al. (22)	2014	Germany	TET2	AML-SHG 0199 & 0295 clinical trial - high-dose cytarabine/daunorubicin (could not get the details)
Tian et al. (23)	2014	China	TET2	158 received 7+3 daunorubicin45 mg/m^2 (Days 1–3) and cytarabine 100 mg/m^2(Days1–7).<br><br>Older patients (>70 years) CAG regimen was used as a first choice consisting of Ara-C10 mg/m^2 injected SC every 12 h (Days1–14), aclarubicin 6 mg/m^2 IV (Days 1-8) and G-CSF 200 mg/m^2/day
Ahn et al. (24)	2015	Korea	TET2	Standard 7+3 (3-day course of anthracycline with a 7-day course of cytosine arabinoside)
Ley et al. (25)	2010	USA	DNMT3a	No details in publication.

LaRochelle et al. (26)	2011	France	DNMT3a	Cytarabine (200mg/m^2) with either daunorubicin (60 mg/m^2 day 1-3) or idarubicin (8 mg/m^2 day 1-5)
Thol et al. (27)	2011	Germany	DNMT3a	AML SHG 0199 & 0295 (no additional details available in publication)
Ribeiro et al. (28)	2012	Netherlands	DNMT3a	HOVON AML protocols HO04, HO04A, HO29, HO42, HO42A, and HO43<br>HO04 and HO04 A no data<br>HO29 cytarabine 200 mg/m^2 for 7 days + idarubicin 12 mg/m^2 for 3 days<br>HO42 cytarabine 200 mg/m^2 for 7 days + idarubicin 12 mg/m^2 for 3 days or cytarabine 1000 mg/m^2 every 12 hours for 10 doses<br>HO42A cytarabine 200 mg/m^2 for 7 days + idarubicin 12 mg/m^2 for 3 days<br>HO43 (patients 61 years and above) cytarabine 200 mg/m^2 for 7 days + daunorubicin 90 mg/m^2 for 3 days vs 45 mg/m^2 for 3 days
Marcucci et al. (29)	2012	USA	DNMT3a	Cytarabine, daunorubicin based (no additional details available in publication)
Renneville et al. (30)	2012	France	DNMT3a	Acute leukemia French Association ALFA-9801 and 9802 trials<br>Induction chemotherapy consists of a timed-sequential chemotherapy including a first sequence of chemotherapy combining daunorubicin, 80 mg/m^2 per day, administered IV as a short infusion over 3 days (days 1-3), and cytarabine, 500 mg/m^2 per day IV as a continuous infusion over the same period. The second sequence, administered after 4-day free interval, consists of mitoxantrone, 12 mg/m^2 per day, administered IV as a short infusion over 2 days (days 8 and 9), and cytarabine, 500 mg/m^2/12h, administered as a 3-hour infusion for 3 days (days 8-10)
Marková et al. (31)	2012	Czechia	DNMT3a	Standard 3+7 Cytarabine + idarubicin or daunorubicin<br>HAM protocol with high dose cytarabine<br>Low dose palliative
Hou et al. (32)	2012	Taiwan	DNMT3a	363 standard induction and cytarabine 100 mg/m^2/d on days 1-7) and then consolidation chemotherapy with 2-4 courses of high dose cytarabine (2000 mg/m^2 every 12 hours on days 1-4 for a total of 8 doses), with or without an anthracycline (idarubicin or Novantrone).<br>143 palliative supportive care or low dose chemotherapy
Gaidzik et al. (33)	2013	Germany	DNMT3a	Treatment protocols of AMLSG<br>AML HD98A two induction cycles of idarubicin, standard-dose cytarabine, and etoposide with or without ATRA, followed by one consolidation cycle of intermediate-dose cytarabine and mitoxantrone with or without ATRA.<br>AMLSG 07-04 two induction cycles of idarubicin, standard-dose cytarabine, and etoposide with or without ATRA, followed by one consolidation cycle of intermediate-dose cytarabine with or without ATRA.

Ostronoff et al. (34)	2013	USA	DNMT3a	SWOG clinical trials S-9031 and S-9333<br><br>S-9031 the patients were randomly assigned to a standard induction regimen (daunorubicin 45 mg/m^2per day for 3 days and Ara-C 200 mg/m^2 per day for 7 days) plus either placebo or G-CSF (400 microg/m^2 once daily). In the S-9333 the patients were randomized to receive mitoxantrone (10 mg/m^2 per day for 5 days) and etoposide (100 mg/m^2 per day for 5 days), or standard induction regimen.
Gale et al. (35)	2015	UK	DNMT3a	AML10, AML12 trials<br><br>AML10 daunorubicin 50 mg/m^2 for three days and twice daily cytarabine for 10 days in course 1 and 8 days in course 2 plus either thioguanine or etoposide<br><br>AML12 cytarabine, daunorubicin and etoposide (ADE) versus mitoxantrone, cytarabine and etoposide (MAE), and compared standard Ara-C dose of 100 mg/m^2 b.i.d. (S-DAT) versus 200 mg/m^2 b.i.d. (H-DAT).
Sehgal et al. (36)	2015	USA	DNMT3a	7 days of Cytarabine + 3 days of anthracycline
Schnittger et al. (37)	2010	Germany	IDH1	1 or 2 induction therapy courses and at least one course of consolidation therapy, each with AraC and an anthracycline
Green et al. (38)	2010	UK	IDH1	AML10, AML12 trials<br><br>AML10 daunorubicin 50 mg/m^2 for three days and twice daily cytarabine for 10 days in course 1 and 8 days in course 2 plus either thioguanine or etoposide<br><br>AML12 cytarabine, daunorubicin and etoposide (ADE) versus mitoxantrone, cytarabine and etoposide (MAE), and compared standard Ara-C dose of 100 mg/m^2 b.i.d. (S-DAT) versus 200 mg/m^2 b.i.d. (H-DAT).
Boissel et al. (39)	2010	France	IDH2	ALFA 9801 and ALFA 9802<br><br>Induction chemotherapy consists of a timed-sequential chemotherapy including a first sequence of chemotherapy combining daunorubicin, 80 mg/m2 per day, administered IV as a short infusion over 3 days (days 1-3), and cytarabine, 500 mg/m^2 per day IV as a continuous infusion over the same period. The second

				sequence, administered after 4-day free interval, consists of mitoxantrone, 12 mg/m^2 per day, administered IV as a short infusion over 2 days (days 8 and 9), and cytarabine, 500 mg/m^2/12h, administered as a 3-hour infusion for 3 days (days 8-10)
Paschka et al. (40)	2010	Germany	IDH	AMLSG trail AML HD98A<br><br>AML HD98A two induction cycles of idarubicin, standard-dose cytarabine, and etoposide with or without ATRA, followed by one consolidation cycle of intermediate-dose cytarabine and mitoxantrone with or without ATRA.
Abbas et al. (41)	2010	Netherlands	IDH1	HOVON AML protocols HO04, HO04A, HO29, HO42, HO42A, and HO43<br>HO04 and HO04 A no data<br>HO29 cytarabine 200 mg/m^2 for 7 days + idarubicin 12 mg/m^2 for 3 days<br>HO42 cytarabine 200 mg/m^2 for 7 days + idarubicin 12 mg/m^2 for 3 days or cytarabine 1000 mg/m^2 every 12 hours for 10 doses<br>HO42A cytarabine 200 mg/m^2 for 7 days + idarubicin 12 mg/m^2 for 3 days<br>HO43 (patients 61 years and above) cytarabine 200 mg/m^2 for 7 days + daunorubicin 90 mg/m^2 for 3 days vs 45 mg/m^2 for 3 days
Abbas et al. (41)	2010	Netherlands	IDH2	HOVON AML protocols HO04, HO04A, HO29, HO42, HO42A, and HO43<br>HO04 and HO04 A no data<br>HO29 cytarabine 200 mg/m^2 for 7 days + idarubicin 12 mg/m^2 for 3 days<br>HO42 cytarabine 200 mg/m^2 for 7 days + idarubicin 12 mg/m^2 for 3 days or cytarabine 1000 mg/m^2 every 12 hours for 10 doses<br>HO42A cytarabine 200 mg/m^2 for 7 days + idarubicin 12 mg/m^2 for 3 days<br>HO43 (patients 61 years and above) cytarabine 200 mg/m^2 for 7 days + daunorubicin 90 mg/m^2 for 3 days vs 45 mg/m^2 for 3 days
Marcucci et al. (42)	2010	USA	IDH	CALGB 9621 AND 19808 for young <60 years<br><br>8525, 8923, 9420, 9720 or 10201 for older >60 years<br><br>CALGB 9621 2 to 5 course of AraC (7 days), DNM (3 days), VP (3 days), or ADE randomized to receive or not receive PSC-8333 (Valspodar)

				CALGB 19808 randomized between 2 induction regimens: Ara-C, Daunorubicin, and Etoposide (ADE) or ADEP with the P-glycoprotein modulator PSC-833 (Kolitz et al, ASH 2005). The remaining 432 patients received ADE induction.
Damm et al. (43)	2011	Germany	IDH	AML-BFM 2004 and AML-BFM 98<br><br>First induction, AIE (cytarabine/idarubicin/ etoposide) or randomized (R1) with ADxE (cytarabine/ L-DNR/etoposide); second induction, (high-dose cytarabine [3g/m 2]/mitoxantrone) (HAM)
Chou et al. (44)	2011	Taiwan	IDH2	309 patients(69.3%) received conventional induction chemotherapy (idarubicin 12 mg/m2per day on days 1–3 and cytarabine 100 mg/m2per day on days 1–7), followed by consolidation chemotherapy with 2–4 cycles of high-dose cytarabine (2000 mg/m2every 12 hon days 1–4, total eight doses) with or without an anthracycline(idarubicin or mitoxantrone) after achieving complete remission(CR)<br><br>137 patients received palliative therapy or low-dose chemotherapy
Nomdedéu et al. (45)	2012	Spain	IDH	CETLAM-03 protocol<br><br>Induction therapy consisted of 1 or 2 courses of idarubicin 12 mg/m^2 IV days 1,3,5, cytarabine 500 mg/m^2/12 h over 2 h IV days 1,3,5,7 and etoposide 100 mg/m^2 IV on days 1, 2 and 3. This was followed by a consolidation phase with mitoxantrone 12 mg/m^2 IV from days 4 to 6, and cytarabine 500 mg/m^2/12 h IV from days 1 to 6. Patients also received G-CSF priming, consisting of 150 mg/m^2 administered subcutaneously (SC) from day 0 to the last day of induction and/consolidation CT.
Ravandi et al. (6)	2012	USA	IDH	The induction regimen in all patients included ara-C 1.5 g/m^2 given by 24 hour continuous infusion daily for 4 days (3 days in patients 60 years or older) and idarubicin 12 mg/m^2 intravenously daily for 3 days. Patients received tipifarnib 300 mg twice daily for 21 days, sorafenib 400 mg twice daily for 7 days, or vorinostat 500 mg 3 times daily for 3 days, with IAT, IAS, or IAV regimens, respectively. Patients could receive up to 2 induction courses
Lin et al. (46)	2012	China	IDH	No details
Guan et al. (47)	2013	China	IDH1	Standard DA or HA regimen
Yamaguchi et al. (7)	2014	Japan	IDH	Patients aged 69 yr or younger were treated with anthracycline and cytarabine according to the protocols of the Japan Adult Leukemia Study Group (JALSG)

				Patients aged 70 yr or older were treated with low-dose cytarabine and aclarubicin in combination with granulocyte colony-stimulating factor
Aref et al. (48)	2015	Egypt	IDH1	All patients received intensive induction therapy (cytarabine 100 $mg/m^2/d$ for 7 days intravenous [I.V.] continuous infusion and daunorubicin90 $mg/m^2/d$ for 3 days I.V.), and consolidation therapy (cytarabine 1 g/m^2 for 12 hours on the first, third, and fifth days with daunorubicin 45 $mg/m^2/d$ for 3 days I.V.).
